# Circadian signatures of adipose tissue in diet-induced obesity

**DOI:** 10.3389/fphys.2022.953237

**Published:** 2022-08-31

**Authors:** Haoran Xin, Jianxin Zhang, Rongfeng Huang, Lihua Li, Sin Man Lam, Guanghou Shui, Fang Deng, Zhihui Zhang, Min-Dian Li

**Affiliations:** ^1^ Department of Cardiovascular Medicine, Center for Circadian Metabolism and Cardiovascular Disease, Southwest Hospital, Third Military Medical University (Army Medical University), Chongqing, China; ^2^ State Key Laboratory of Molecular Developmental Biology, Institute of Genetics and Developmental Biology, Chinese Academy of Sciences, Beijing, China; ^3^ Lipidall Technologies Company Limited, Changzhou, China; ^4^ Department of Pathophysiology, College of High Altitude Military Medicine, Army Medical University (Third Military Medical University), Chongqing, China

**Keywords:** circadian clock, high-fat diet, adipose tissue, transcriptomics, CLOCK, CES1D, lipidomics

## Abstract

High-fat diet (HFD) feeding rewires circadian rhythms of peripheral organs including the liver and adipose tissue. While the liver has been extensively studied, it remains largely unknown whether and how HFD organizes circadian biology in adipose tissue. Here, we took a systems approach to profile the diurnal transcriptome of adipose tissue in diet-induced obese mice either fed a low-fat diet (LFD) that reduces weight or still fed HFD. We detected about 200 and 2,500 diurnal genes in HFD and LFD, respectively. Pathway analysis revealed that rhythmic pathways in HFD are represented by circadian rhythm, ribosome biogenesis, and nucleosome organization, whereas those in LFD are represented by myeloid cell function. Remarkably, the majority of the circadian clock genes, except *Clock,* exhibited robust diurnal rhythm in the adipose tissue of HFD-fed mice. Analysis of mRNAs and proteins in another cohort of HFD-fed mice confirmed that *Clock* lost rhythmicity at the transcript, but not protein level. Diet reversal to LFD specifically restored diurnal difference of the *Clock* transcripts in adipose tissue. We matched transcriptomics data with global profiling of neutral lipids and found that lipid metabolism catalyzed by triglycerol hydrolase *Ces1d* is a key circadian feature that is activated by diet reversal. Together, our work defines the circadian signatures in the adipose tissue of diet-induced obese mice, and their flexibility upon dietary intervention, thereby shedding light on potential clock-modulated tissue-specific pathways during obesity.

## Introduction

It is well-recognized that circadian dysfunction underlies the pathogenesis of metabolic diseases linked to high calorie intake (Asher and Sassone-Corsi, 2015; Allada and Bass, 2021; Guan and Lazar, 2021; López-Otín and Kroemer, 2021). High-fat diet (HFD) feeding disrupts the diurnal rhythm of food intake and has pathogenic consequences, which can be prevented by time-restricted feeding or restricted feeding in the active phase of the day (Chaix et al., 2019; Li, 2022). Conversely, inverted feeding, when combined with HFD, may contribute to increased adiposity and glucose intolerance in mice (Arble et al., 2009; Bray et al., 2013; Zhang et al., 2020). HFD can decrease the amplitude of peripheral clocks in adipose tissue and liver, and the adipose clock seems more susceptible to dampening than the liver clock (Kohsaka et al., 2007). The landscape of circadian reprogramming in the liver has been well-characterized. HFD impairs the chromatin binding capacity of core clock transcription factors and generates *de novo* oscillation in the transcriptome through metabolic nuclear receptors, including peroxisome proliferator-activated receptors (Eckel-Mahan et al., 2013; Murakami et al., 2016; Guan et al., 2018). We and others have recently shown that peripheral organs exhibit distinct capacity to adapt to inverted feeding with respect to the regulation of circadian rhythm in each organ (Xin et al., 2021a; Manella et al., 2021). Thus, it is necessary to probe whether and how adipose tissue reorganizes circadian rhythms in HFD feeding and the dynamics of circadian rhythms upon the reversal to a low-fat diet (LFD).

## Methods

### Animal studies

C57BL/6J male mice (Hunan SJT Laboratory Animal Co., *n* = 12) were maintained under a 12 h/12 h light/dark cycle under constant temperature (22°C) and humidity, with free access to regular chow diet and water. After acclimation, male mice at the age of 8 weeks were fed a high-fat diet (HFD, 60 kcal% fat, Research Diets Inc. D12492) for 13 weeks. Mice were then randomly assigned to one of the two groups, as described previously (Xin et al., 2021b); one group was fed HFD for 7 days and the other was fed a matched low-fat diet (LFD, 10 kcal% fat 7% sucrose, sucrose matched to HFD, Research Diets Inc. D12450J). Weight loss and energy intake were measured on the first, third, and sixth day, as described previously (Li et al., 2018). Epididymal adipose tissue was dissected from mice at Zeitgeber time 0 h (9:00 a.m., ZT0, the light-on time) and ZT12 (the light-off time, *n* = 3 biological replicates), snap-frozen in liquid nitrogen, and stored at −80°C. HFD-fed obese mice for validation experiments were raised on normal chow diet until 19 weeks of age and then fed HFD for 30 weeks. All procedures were approved by the Laboratory Animal Welfare and Ethics Committee of the Third Military Medical University (TMMU), China.

### RNA sequencing

Total RNA was extracted using the TRIzol method (Invitrogen). RNA integrity, purity, and concentration were assessed using RNA electrophoresis, the NanoPhotometer spectrophotometer, and the Bioanalyzer 2100 system (Thermo Fisher Scientific, MA, United States). All RNA samples passed quality control. For each experiment, 100 mg adipose tissue was subjected to homogenization (*n* = 3 biological replicates). Ribosomal RNA was removed using the Epicenter Ribo-Zero Kit (Illumina, CA, United States). Library construction, next-generation RNA sequencing, read mapping, and gene quantification were performed by Novogene (Beijing, China), as described previously (Xin et al., 2021a). Library preparations were sequenced on an Illumina NovaSeq6000 platform and 150 bp paired-end reads (PE150) were generated. Clean reads were obtained by removing reads containing adapter, reads containing poly-N, and low-quality reads from raw data. All the downstream analyses were based on high quality clean data. HISAT2 v2.0.5 was used to index the reference genome (mus_musculus_Ensembl_97) and map the paired-end clean reads. Transcripts were assembled and quantified as read counts and tags per million (TPM) by StringTie (Pertea et al., 2015). Correlations among RNA-seq samples were assessed using Pearson correlation coefficients.

### Gene expression analysis

Tissue RNA was isolated using Eastep Super Total RNA Extraction Kit (Promega). Complementary DNA (cDNA) was synthesized using the GoScript Reverse Transcription Mix (Promega). cDNA was amplified and analyzed using iTaq universal SYBR Green Supermix (Bio-Rad) and the Bio-Rad CFX96 Real-Time PCR Detection System (Bio-Rad). PCR protocol: Polymerase activation and DNA denaturation at 95°C for 3 min; denaturation at 95°C for 10 s; annealing/extension and plate read at 60°C for 30 s; 40 cycles of quantitative PCR. Results were quantified using a standard curve and normalized to u36B4 (Rplp0). Experiments were repeated at least twice. Primer sequences are as follows. Bmal1-F, GAA​AAG​AGG​CGT​CGG​GAC​AA; Bmal1-R, GCC​ATC​CTT​AGC​ACG​GTG​AG; Clock-F, AAG​GCA​TGT​CAC​AGT​TTC​AGT​T; Clock-R, CTC​TAT​CAT​CCG​TGT​CCG​CTG; Dbp-F, CGT​GGA​GGT​GCT​AAT​GAC​CTT​T; Dbp-R, CAT​GGC​CTG​GAA​TGC​TTG​A; Lep-F, GAG​ACC​CCT​GTG​TCG​GTT​C; Lep-R, CTG​CGT​GTG​TGA​AAT​GTC​ATT​G; Nr1d1-F, TAC​ATT​GGC​TCT​AGT​GGC​TCC; Nr1d1-R, CAG​TAG​GTG​ATG​GTG​GGA​AGT​A; Nr1d2-F, TGA​ACG​CAG​GAG​GTG​TGA​TTG; Nr1d2-R,GAGGACTGGAAGCTATTCTCAGA; Per1-F, TGA​AGC​AAG​ACC​GGG​AGA​G; Per1-R, CAC​ACA​CGC​CGT​CAC​ATC​A; Per2-F, ATG​CTC​GCC​ATC​CAC​AAG​A; Per2-R, GCG​GAA​TCG​AAT​GGG​AGA​AT; Rpl36-F, GGC​CAC​AAG​GTG​ACG​AAA​AAC; Rpl36-R, GCG​CTT​GCT​CTT​GGA​CAC​T; Tef-F, CCT​GCT​GGA​GCA​TTC​TTT​GC; Tef-R, ATC​GTA​GGG​GAT​GGT​CTT​GTC; u36B4-F, AGA​TGC​AGC​AGA​TCC​GCA​T; u36B4-R, GTT​CTT​GCC​CAT​CAG​CAC​C.

### Targeted profiling of neutral lipids

Lipid extracts from adipose tissue were prepared using a modified Bligh/Dyer method and analyzed as described previously (Xin et al., 2021a; Lam et al., 2021). Samples were resuspended and spiked with appropriate concentrations of isotope-labeled internal standards. All lipidomics analyses were conducted on an Exion UPLC coupled with a SCIEX QTRAP 6500 PLUS system in ESI mode (curtain gas = 20, ion spray voltage = 5500 V, temperature = 400°C, ion source gas 1 = 35, ion source gas 2 = 35). Glycerolipids including diacylglycerols (DAGs) and triacylglycerols (TAGs) were quantified using a modified version of reverse phase LC-MRM-MS. Separation of neutral lipids was achieved on a Phenomenex Kinetex-C18 2.6 µm column (i.d. 4.6 mm × 100 mm) using an isocratic mobile phase containing chloroform:methanol:0.1 M ammonium acetate at 100:100:4 (v/v/v), respectively, at a flow rate of 160 μl/min. Levels of TAGs were quantified by referencing to spiked internal standards of TAG (14:0)3-d5, TAG (16:0)3-d5, and TAG (18:0)3-d5 obtained from CDN Isotopes, Inc. (Quebec, Canada). DAGs were quantified using d5-DAG (1,3-17:0) and d5-DAG (1,3-18:1) as internal standards from Avanti Polar Lipids (AL, United States). Free cholesterol and cholesteryl esters were analyzed by HPLC-MS/MS in APCI mode, and estimated by referencing to internal standards, i.e., cholesterol-26,26,26,27,27,27-d6 and cholesteryl-2,2,3,4,4,6-d6 Octadecanoate (CDN Isotopes, Inc.). Lipid levels were expressed as micromole of lipids per gram of total protein.

### Acyl-CoA quantification by LC/MS

Acyl-CoA was extracted from adipose tissue and quantified as described (Lam et al., 2020; Xin et al., 2021a). Briefly, 300 µl of extraction buffer containing isopropanol, 50 mM KH_2_PO_4_, 50 mg/ml bovine serum albumin (25:25:1 v/v/v) acidified with glacial acetic acid was added to samples. As an internal standard, 19:0-CoA was added and the sample mix was homogenized. Next, 300 µl of petroleum ether were added and the sample was centrifuged at 12,000 rpm for 2 min at 4°C. The upper phase was removed. The extraction process was repeated two more times with petroleum ether. Next, 5 µl of saturated ammonium sulfate were added to the lower phase, followed by 600 µl of chloroform:methanol (1:2 v/v). The sample was then incubated on a thermomixer at 450 rpm for 20 min at 25°C, followed by centrifugation at 12,000 rpm for 5 min at 4°C. Clean supernatant was transferred to a fresh tube and subsequently dried in a SpeedVac in OH mode (Genevac). Dry extracts were resuspended in an appropriate volume of methanol:water (9:1 v/v) prior to liquid chromatography–mass spectrometry (LC–MS) analyses on a Thermofisher U3000 DGLC coupled to Sciex QTRAP 6500 Plus.

### Differential expression analysis

Differential expression (DE) analysis between (1) HFD ZT0 vs HFD ZT12, (2) LFD ZT0 vs HFD ZT0, (3) LFD ZT12 vs HFD ZT12, and (4) LFD ZT0 vs LFD ZT12 was performed using the DESeq2 R package (1.10.1), which is based on the negative binomial distribution. The resulting *p*-values were adjusted using the Benjamini and Hochberg’s approach (mRNA) or Tukey’s HSD method (lipid) for controlling the false discovery rate. Protein-coding genes (annotated as protein-coding by Ensembl) and lipids with an adjusted *p*-value of less than 0.05 were considered differentially expressed genes (DEGs) and differentially regulated lipids, respectively.

### Gene ontology pathway analysis of differentially expressed genes

We used clusterProfiler (4.0.5) R package to test the statistical enrichment of DEGs. Considering the small number of DEGs, gene set enrichment analysis (GSEA) using Gene Ontology (GO) terms was conducted for DEGs. GSEA was implemented through the gseGO function of the clusterProfiler (4.0.5) R package using the default settings. GO terms with an adjusted *p*-value of less than 0.05 were considered significantly enriched DEGs. The results were plotted and further analyzed by hierarchical clustering using enrichplot (1.12.3) R package.

### Multi-omics N-integrative supervised analysis

Integrative analysis of the adipose transcriptome and global profiles of neutral lipids was performed using the DIABLO (Data Integration Analysis for Biomarker discovery using Latent variable approaches for Omics studies) model in the R package mixOmics (6.18.1) (Rohart et al., 2017). First, omics features were selected using sparse Partial Least Squares Discriminant Analysis (sPLS-DA). Next, 43 differentially regulated lipids and 109 DEG features were selected for two data blocks (lipid and mRNA), respectively. The following modeling parameters were used after tuning: ncomp = 3 (number of model components); the number of lipids to consider: 40, 8; the number of mRNAs to consider: 8, 8, 60. Data were then modeled in the DIABLO framework. Plots of experimental groups (function: plotIndiv) provide a visual representation of samples in the DIABLO framework. Plots of variables (function: plotVar), Correlation Circle plot (function: circosPlot), and plots of variable loadings (importance of the feature/molecule) were produced in the mixOmics R package (6.16.3).

### Immunoblot analysis

Adipose tissues were lysed in RIPA buffer supplemented with proteinase inhibitors and protein phosphatase inhibitors. 30 µg of protein lysate were electrophoresed on SDS-PAGE gels at 120 V and transferred to activated PVDF membranes (Bio-Rad #1620177). The membranes were blocked in 5% skim milk for 1 h and incubated with primary antibodies at 4°C overnight. CLOCK (Abclonal, A7265, rabbit monoclonal, 1:1,000); FABP4 (Abclonal, A11481, rabbit monoclonal, 1:1,000). Immunoblotting was visualized using peroxidase-conjugated secondary antibodies, enhanced chemiluminescent substrate (Bio-Rad #1705062), and the Azure C500 imaging system. Experiments were repeated at least twice (*n* = 2 biological replicates per time point).

### Statistical analysis

Statistical significance was determined by one-way analysis of variance (ANOVA) and using the two-tailed *t*-test for comparison of two groups in R (4.1.3) and RStudio (2022.02.3 + 492). Circadian rhythmicity was determined by RAIN (1.26.0), Circacompare (0.1.1), and MetaCycle (1.2.0) (Thaben and Westermark, 2014; Wu et al., 2016; Parsons et al., 2020). Values of *p* < 0.05 or adjusted *p* < 0.05 indicated statistical significance. The results were plotted using R packages such as ggpubr (0.4.0), ggplot2 (3.3.5), and pheatmap (1.0.12).

## Results

### Identification of transcripts and lipids altered by diet reversal and time of day in adipose tissue of diet-induced obese mice

**FIGURE 1:**
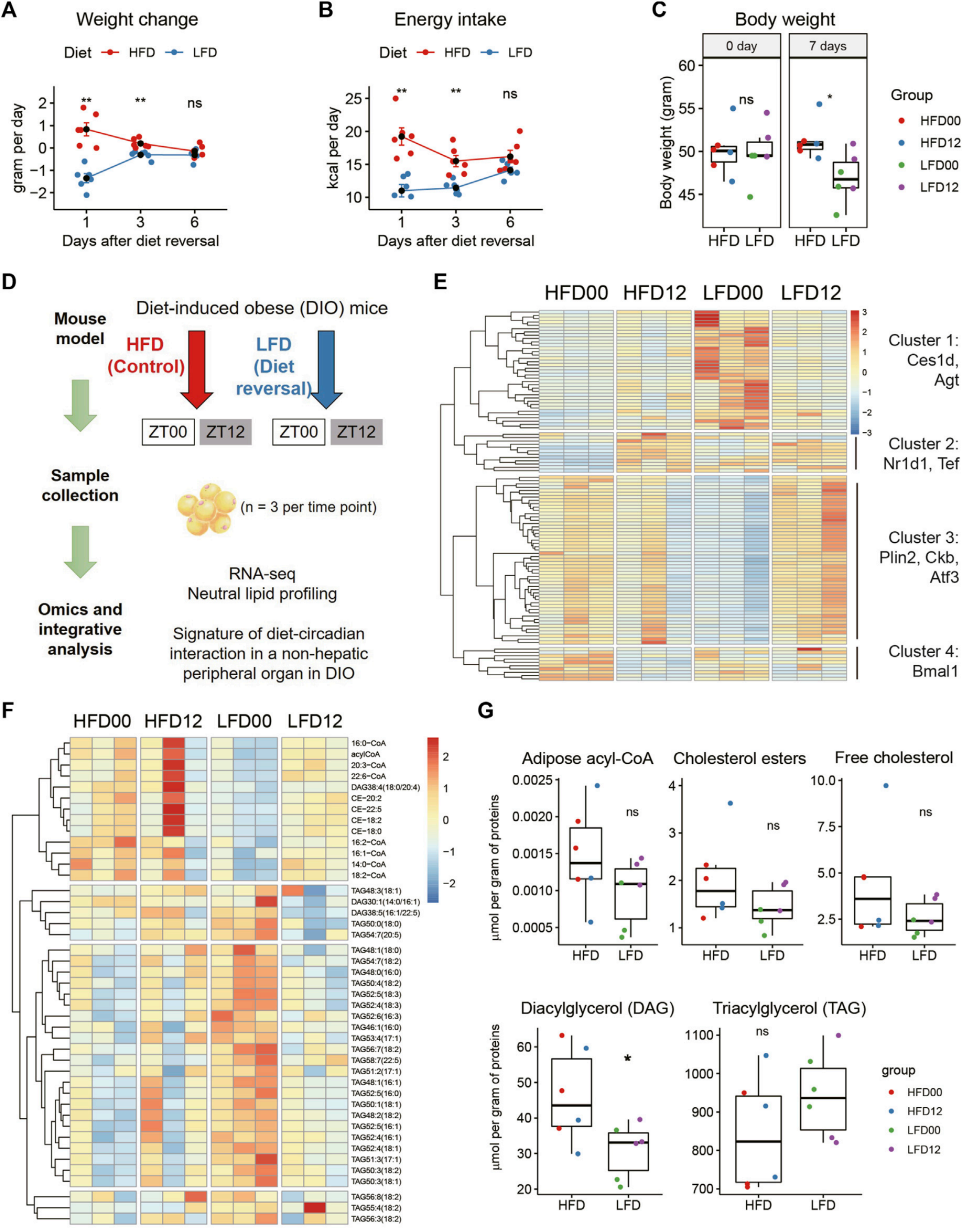
Identification of transcripts and lipids altered by diet reversal and time of day in adipose tissue of diet-induced obese mice. Twelve male DIO mice were randomly assigned to high-fat diet (HFD, Control) and low-fat diet (LFD, Diet-reversal) for 7 days. **(A–C)** Weight change **(A)**, energy intake **(B)**, and body weight **(C)** before and after 7 days of diet reversal. **(D)** Study design. Mice were sampled at ZT0 and ZT12. VAT samples were also subjected to RNA-seq and quantitative mass spectrometry analyses for neutral lipids and acyl-CoAs. **(E)** Representative differentially expressed genes from pairwise comparisons between the four groups (adjusted *p*-value < 0.05). **(F)** Differentially regulated lipids in VAT (adjusted *p*-value < 0.05, Tukey’s HSD test). **(G)** Total concentrations of lipid species by class in VAT. DIO, diet-induced obese; VAT, visceral adipose tissue; ZT, zeitgeber time. HFD00, HFD mouse samples collected at ZT0; HFD12, HFD samples collected at ZT12; LFD00, LFD mouse samples collected at ZT0; LFD12, LFD samples collected at ZT12. Data are represented as mean ± SEM and jittered points. One-way ANOVA and unpaired *t*-test, *p < 0.05.

To probe this question, we generated a mouse model of diet reversal. Twelve C57BL/6J male mice were fed HFD for 13 weeks and achieved an average body weight of 50 g. These obese mice were each randomly assigned to one of two groups. The diet reversal group was put on LFD for 7 days, and the control group stayed on HFD for 7 days (*n* = 6 mice). We tracked energy metabolism and found that LFD mice lost the most weight on day 1 (−1.35 ± 0.18 versus 0.83 ± 0.27 g per day, *p* = 0.000642), followed by moderate weight loss on day 3 (Figure 1A). Weight loss became indistinguishable between the two groups on day 6 (Figure 1A). This is associated with a similar pattern of energy intake (Figure 1B). Thus, by the end of the treatment period, LFD mice reached a steady state in the rate of weight change and achieved 8.16% reduction in body weight, compared to HFD mice (LFD, −5.79 ± 1.39%; HFD, 2.37 ± 1.79%, *p* = 1.59E-05, Figure 1C). This percentage of weight loss is clinically relevant, as it is within the 5%–10% weight loss range prescribed for a patient with metabolic disease.

We sampled epidydimal adipose tissue (also known as visceral adipose tissue [VAT] in mice) at ZT0 and ZT12, respectively (*n* = 3 mice, Figure 1D). These samples were subjected to RNA sequencing for a depth of 40 million reads, as recommended based on a prior report (Hughes et al., 2017). We applied a filter of fold change (FC), i.e., absolute log2FC between 1 and 2, to reveal robust DEGs. After pair-wise comparison for dietary and circadian effects, we detected 109 robust DEGs in VAT (Figure 1E). Hierarchical clustering identified four clusters of DEGs. The first cluster showed high expression of genes in the LFD00 group, including triglycerol hydrolase (*Ces1d*) and angiotensinogen (*Agt*). The second cluster showed low expression of genes in the HFD00 group, including some circadian clock genes (*Nr1d1, Tef*). The third cluster showed low expression of genes in the LFD00 group, including lipid droplet coat protein *Plin2*, creatine kinase b (*Ckb*) and transcription factor *Atf3*. The fourth cluster included *Bmal1 (Arntl)*.

VAT samples were further subjected to quantitative profiling of neutral lipids and acyl coenzyme As (acyl-CoAs). We detected 43 differentially regulated lipids after pairwise comparisons between groups at an adjusted *p*-value of 0.05 (Figure 1F). These lipids span in class from acyl-CoA and cholesterol ester to diacylglycerol and triglycerol. We did not find statistically significant differences in total acyl-CoA, cholesterol ester, free cholesterol, or triglycerol, but diacylglycerol was significantly lower in mice that received LFD versus HFD (Figure 1G).

### Effects of high-fat diet and diet reversal on the diurnal transcriptome in adipose tissue

**FIGURE 2:**
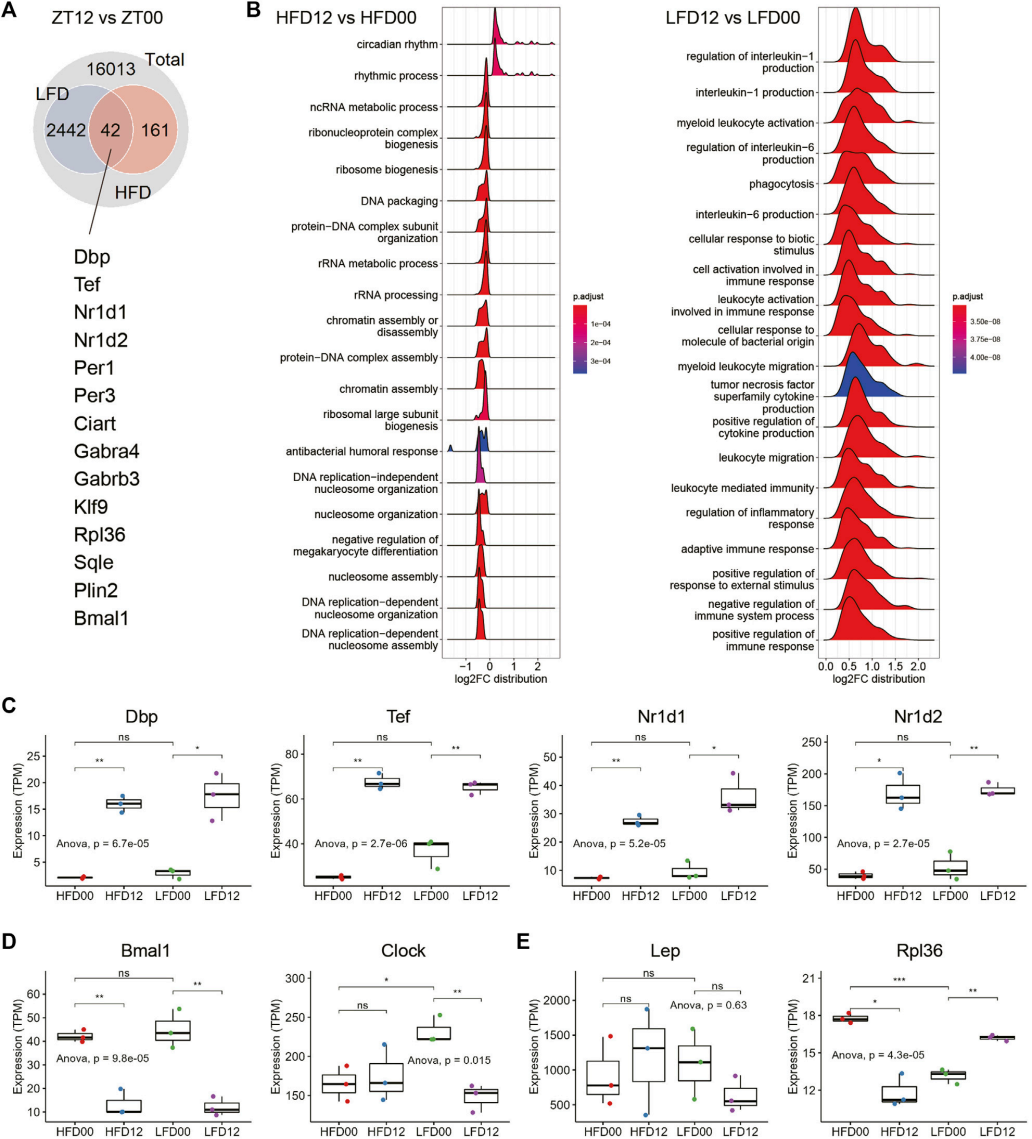
Effects of high-fat diet and diet reversal on the diurnal transcriptome in adipose tissue. **(A)** Interaction of DEGs between LFD12-vs-LFD00 and HFD12-vs-HFD00. **(B)** Ridge plots showing the results of gene set enrichment analysis (GSEA) of differentially expressed genes (DEGs) between ZT12 and ZT0 in VAT of HFD (left panel) and LFD (right panel) mice. **(C)** Expression of representative circadian clock genes in VAT. **(D)** Expression of *Bmal1* and *Clock* genes in VAT. **(E)** Expression of genes related to adipokine and ribosome biogenesis. VAT, visceral adipose tissue. HFD00, HFD mouse samples collected at ZT0; HFD12, HFD samples collected at ZT12; LFD00, LFD mouse samples collected at ZT0; LFD12, LFD samples collected at ZT12. Data are represented as mean ± SEM and jittered points. One-way ANOVA and unpaired *t*-test, *p < 0.05, **p < 0.01.

Next, we sought to determine circadian signatures in VAT and the interaction of these signatures in the context of HFD and LFD. DEGs between ZT12 and ZT0 are considered circadian or diurnal. At an adjusted *p*-value of 0.05, there were more than 2,400 diurnal genes in the LFD group, and only 203 in the HFD group (Figure 2A). There were 42 diurnal genes in common between the LFD and HFD groups. Notably, these shared DEGs include many components of the circadian clock, including *Dbp, Tef, Nr1d1, Nr1d2*, etc. GSEA revealed that circadian rhythm, ribosome biogenesis, and nucleosome organization and assembly represent major biological processes that are differentially regulated between ZT12 and ZT0 in the HFD group (Figure 2B). Although a considerable number of circadian clock genes are diurnal in the VAT of LFD mice, GSEA showed that the most enriched diurnal pathways in the LFD group are related to myeloid leukocyte biology and production of proinflammatory cytokines (Figure 2B). This transcriptomic signature may indicate that the diurnal activity of the adipose tissue immune system may be involved in the adaptation to diet reversal during obesity.

We surveyed expression profiles of the circadian clock in VAT and found that clock genes, such as *Dbp, Tef, Nr1d1,* and *Nr1d2*, exhibited robust diurnal differences in both HFD and LFD groups (Figure 2C). Components of the bipartite core clock transcription factor, i.e., *Clock* (Circadian locomotor output cycles kaput) and *Bmal1* (Brain and muscle Arnt-like 1) showed different expression patterns in VAT. While *Bmal1* mRNA remained diurnal in both the HFD and LFD groups, *Clock* mRNA levels did not show a diurnal pattern in the HFD condition (Figure 2D). Notably, diet reversal to LFD restored the diurnal pattern in *Clock* mRNA. Leptin (*Lep*) mRNA expression did not exhibit significant differences in either the HFD or LFD groups (Figure 2E). Ribosomal protein L36 (*Rpl36*) is a DEG found in the HFD group. The diurnal pattern of *Rpl36* mRNA expression in the HFD group was a reverse of that in the LFD group (Figure 2E). Overall, the transcriptomic signature in the HFD group indicates that the circadian clock exhibits robust rhythmic activity in the VAT of obese mice, except for some genes, such as *Clock*. These data also indicate that ribosome biogenesis, as represented by expression of *Rpl36*, may exhibit diurnal rhythm in the VAT of obese mice in a diet-dependent manner.

### Clock protein exhibits robust rhythm despite loss of transcript rhythm in the adipose tissue of obese mice

**FIGURE 3:**
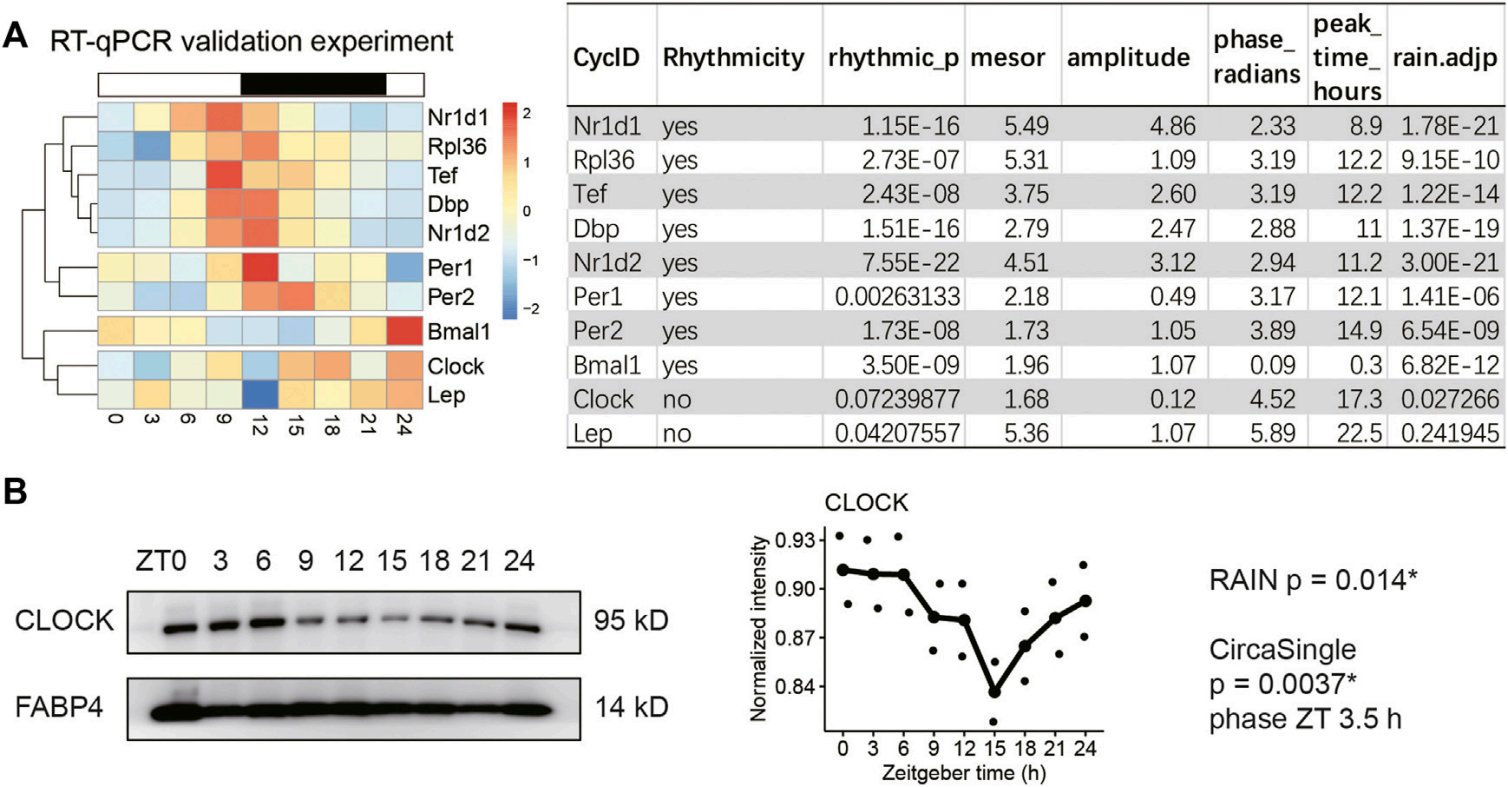
CLOCK protein exhibits robust rhythm despite loss of transcript rhythm in the adipose tissue of obese mice. Visceral adipose tissue (VAT) samples were collected every three hours for a complete 24-hour diurnal cycle in two different cohorts of male diet-induced obese mice. Mice had been fed HFD for 30 weeks. **(A)** Realtime quantitative PCR analysis of selected genes in VAT (*n* = 2 mice per time point for nine time points, repeated three times). **(B)** Representative immunoblots of CLOCK and FABP4 (loading control) proteins in the VAT from male obese mice (*n* = 2 mice per time point for nine time points, repeated twice). Rhythmicity was determined by RAIN and CircaCompare R packages for a threshold adjusted *p*-value of 0.05 and 0.01, respectively. Data are represented as mean and jittered points.

Next, we sought to validate the expression profiles of the circadian clock and selected genes involved in adipokine production (*Lep*) and ribosome biogenesis (*Rpl36*) in obese mice that had been sampled every three hours for a complete diurnal cycle. The representative clock genes are from three major feedback loops, including those shown in Figures 2C,D and *Per1/2*. The 24-hour expression profiles were subjected to rhythmicity analysis using two different algorithms, including RAIN and circa_single (available in the R package: Circacompare). The genes that reached statistical significance (adjusted *p*-value < 0.05 in RAIN and 0.01 in circa_single) in both algorithms were considered as rhythmic transcripts. As shown in Figure 3A, all clock genes exhibited robust diurnal rhythms in VAT of HFD-fed mice except *Clock* mRNA. Indeed, hierarchical clustering analysis segregated *Clock* from *Bmal1*, supporting distinct daily patterns between *Clock* and *Bmal1* (Figure 3A). Adipose tissue *Rpl36* exhibited diurnal rhythm in transcript levels, whereas *Lep* mRNA did not, which is consistent with the transcriptome profiling results (Figure 2E).

Immunoblotting showed that CLOCK protein cycled robustly in the adipose tissue of HFD-fed mice (Figure 3B, RAIN *p* = 0.014*, circa_single *p* < 0.01, phase ZT3.5). Thus, we have validated the diurnal expression of the circadian clock, *Rpl36,* and *Lep* in the adipose tissue of obese mice. Although *Clock* mRNA is arrhythmic, CLOCK protein maintains robust diurnal oscillation in the adipose tissue of obese mice.

### Multi-omics N-integrative supervised analysis in adipose tissue

**FIGURE 4:**
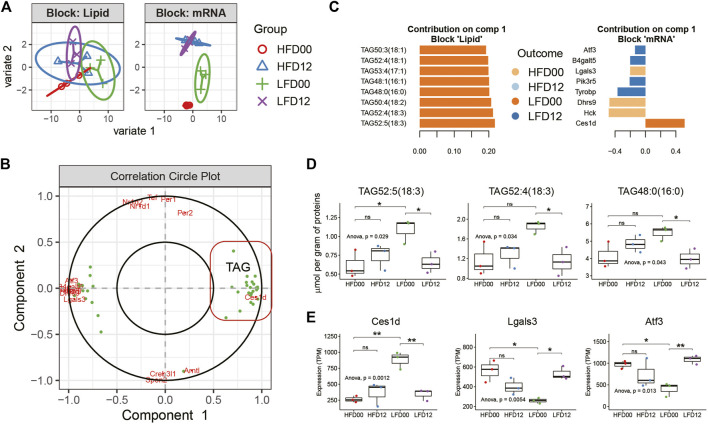
Multi-omics N-integrative supervised analyses in adipose tissue. **(A)** Clustering analysis of samples per data set. **(B)** Correlation Circle plot representing each type of selected features. Correlation coefficient > 0.6. **(C)** Loading plot of each feature selected on component 1 in each omics study, the color of which indicates the class with a maximal mean expression value. **(D)** Tissue concentration of representative lipid species in component 1. **(E)** Expression profiles of representative transcripts in component 1. Data are represented as mean ± SEM and jittered points, one-way ANOVA and unpaired *t*-test, *p < 0.05, **p < 0.01. TAG, triacylglycerol.

To elucidate features of the diet-circadian interaction network in the VAT of obese mice, we performed transcript-lipid network integration. Expression profiles of DEGs and differentially regulated lipids arising from pairwise comparisons between the four experimental groups served as input data for Sparse Partial Least Squares Discriminant Analysis (sPLS-DA) using mixOmics R package. With the tuned parameters for each omics data set, we integrated profiles of transcripts and lipids for N-integration supervised analysis. Clustering analysis clearly segregated the groups based on differentially expressed genes (Figure 4A). Correlation analysis identified two components. Component 1 included *Ces1d* and triacylglycerols (TAGs), and component 2 was largely composed of the circadian clock genes (Figure 4B). By examining the importance plots of component 1, we found that both TAGs and *Ces1d* contributed positively to the LFD00 group, whereas other DEGs, including Activating transcription factor 3 (*Atf3*) and Galectin 3 (*Lgals3*) contributed to other groups (Figure 4C). Representative lipids, such as TAG52:5 (18:3), TAG52:4 (18:3), and TAG48:0 (16:0), exhibited a diurnal difference in the LFD group, but not in the HFD group (Figure 4D). These results matched the diurnal expression pattern of *Ces1d* mRNA (Figure 4E). *Lgals3* and *Atf3* exhibited diurnal variation in the LFD group, but the pattern was the reverse of that of *Ces1d*. Thus, integrative analysis revealed two key circadian features associated with the VAT of HFD-fed mice, including *Ces1d*-related TAG metabolism that is conditionally diurnal upon diet reversal, and a circadian clock that exhibits robust diurnal oscillation in both HFD and LFD conditions.

## Discussion

Our work using transcriptomics and global lipid profiling has revealed circadian signatures of adipose tissue from diet-induced obese animals. We also tested the flexibility of the adipose tissue transcriptome and lipidome upon reversal to a nutritionally balanced control diet. Circadian rhythm, ribosome biogenesis, and nucleosome assembly represent key circadian features in the adipose tissue of obese mice. We have further demonstrated that the majority of the adipose tissue clock genes remain as robustly rhythmic in HFD feeding as they are in diet reversal to LFD. The *Clock* gene is an exception in that HFD disrupts the diurnal rhythmicity of *Clock* mRNA in adipose tissue, which is restored by diet reversal to LFD for 7 days and associated with a clinically meaningful 8% loss of body weight. It is known that diurnal rhythms of the adipocyte clock genes, such as *Clock, Bmal1,* and *Per2*, are dampened during a short-term 6-week HFD feeding (Kohsaka et al., 2007). The adipose circadian clock is dampened upon inverted feeding within 7 days in both male and female mice (Xin et al., 2021a), and this dampening effect lasts for as long as a month in males (Manella et al., 2021). While circadian disruption by short-term HFD or inverted feeding seems to be detrimental to the adipose circadian clock, our findings suggest that the clock may restore robust rhythm with long-term HFD feeding (>12 weeks).

Transcript-network integrative analysis has identified a subset of triglyceride species and the *Ces1d* gene as a crucial feature with rhythms that could be induced by diet reversal to LFD. CES1D is emerging as a crucial clock-modulated triglyceride lipase in adipose tissue during obesity. Adipocyte-specific deletion of *Ces1d* has been shown to increase susceptibility to diet-induced obesity and impaired adaptive thermogenesis (Yang et al., 2019; Li et al., 2022). Additionally, *Ces1d* knockout leads to hypertrophy of adipocytes (Wei et al., 2010). Mechanistically, adipocyte CES1D protein translocates to the surface of lipid droplets upon beta-adrenergic stimulation or cold exposure and facilitates lipolysis and release of non-esterified polyunsaturated fatty acids (Yang et al., 2019). It is transcriptionally upregulated in human diseases, such as obesity and type 2 diabetes (Li et al., 2022), and the circadian rhythm of *Ces1d* expression (peak time ZT14-20) has been observed in many organs from lean mice, including the heart, kidneys, liver, and muscle (Zhang et al., 2014). However, *Ces1d* mRNA expression does not exhibit circadian rhythm in the adipose tissue of lean mice fed *ad libitum* or time restricted diets, despite containing E-box elements in the promoter region of the gene (Zhang et al., 2014; Xin et al., 2021a). It has been shown that adipocyte *Bmal1* knockout reduces *Ces1d* mRNA in adipose tissue from obese mice at ZT6, which is linked to lower concentration of polyunsaturated fatty acids (Paschos et al., 2012). Our results suggest that diet reversal from HFD to LFD induces diurnal rhythm of *Ces1d* in adipose tissue (Figure 4E), as well as diurnal rhythms of a panel of triglyceride species (Figure 4D). Thus, CES1D and its triglycerol hydrolase activity may be controlled by the adipocyte clock during diet reversal and gauge excess fatty acid flux into the circulation and ectopic organs.

This study has several limitations. First, it covers only two time points within 1 day. We may have missed many features that peak in the middle of the diurnal cycle. Future studies may cover two cycles with frequent sampling intervals (e.g., 2 h) to uncover fine details. Second, post-translational regulation is emerging as a crucial component of circadian rhythm in organ systems (Robles et al., 2017; Brüning et al., 2019; Noya et al., 2019; Hansen et al., 2021). It will be necessary to apply proteomics and flux analysis to define physiological outcomes of circadian rhythm in the context of obesity. Third, we have not explored the responsiveness of adipocyte clock output pathways, also known as clock-controlled checkpoints (Li, 2022), towards time-restricted eating and other chronobiological intervention, which would shed light on the translational potential of these pathways.

## Data Availability

The datasets presented in this study can be found in online repositories. The names of the repository/repositories and accession number(s) can be found below: https://db.cngb.org/search/project/CNP0002967/.
